# Impact of cardiovascular risk on the diagnostic accuracy of the ultrasound Halo Score for giant cell arteritis

**DOI:** 10.1186/s13075-022-02920-9

**Published:** 2022-10-13

**Authors:** Juan Molina-Collada, Katerine López Gloria, Isabel Castrejón, Juan Carlos Nieto-González, Julia Martínez-Barrio, Ana M. Anzola Alfaro, Javier Rivera, José María Álvaro-Gracia

**Affiliations:** 1 Department of Rheumatology, Hospital General Universitario Gregorio Marañón, Calle del Dr. Esquerdo, 46, 28007 Madrid, Spain; 2 Instituto de Investigación Sanitaria Gregorio Marañón (IiSGM), Madrid, Spain

**Keywords:** Ultrasound, Halo Score, Giant cell arteritis, Cardiovascular risk, Vasculitis

## Abstract

**Objective:**

To evaluate the impact of cardiovascular risk (CVR) on the diagnostic accuracy of the ultrasonographic (US) Halo Score in patients with suspected giant cell arteritis (GCA).

**Methods:**

Retrospective observational study of patients referred to our US fast track clinic with suspected GCA for a 2-year period. The intima-media thickness (IMT) of cranial and extra-cranial arteries and the Halo Score was determined to assess the extent of vascular inflammation. The European Society of Cardiology Guidelines on CV Disease Prevention were used to define different categories of CVR and patients were classified according to the Systemic Coronary Risk Evaluation (SCORE). The gold standard for GCA diagnosis was clinical confirmation after a 6-month follow-up.

**Results:**

Of the 157 patients included, 47 (29.9%) had GCA after a 6-month follow-up. Extra-cranial artery IMT was significantly higher in patients with high/very high CVR than in those with low/moderate CVR, but only among patients without GCA. Non-GCA patients with high/very high CVR had also a significantly higher Halo Score in contrast with low/moderate CVR [9.38 (5.93) vs 6.16 (5.22); *p* = 0.007]. The area under the ROC curve of the Halo Score to identify GCA was 0.835 (95% CI 0.756–0.914), slightly greater in patients with low/moderate CVR (0.965 [95% CI 0.911–1]) versus patients with high/very high CVR (0.798 [95% CI 0.702–0.895]). A statistically weak positive correlation was found between the Halo Score and the SCORE (*r* 0.245; *c* = 0.002).

**Conclusions:**

Elevated CVR may influence the diagnostic accuracy of the US Halo Score for GCA. Thus, CVR should be taken into consideration in the US screening for GCA.

## Introduction

Giant cell arteritis (GCA) is the most common form of large vessel vasculitis [1] and a potentially life-threatening inflammatory condition. When suspected, GCA requires prompt clinical evaluation and fast diagnostic imaging tests for an early and accurate diagnosis [2]. Due to its high specificity, the gold standard for its diagnosis has traditionally been considered a temporal artery (TA) biopsy. However, ultrasound (US) has emerged as a rapid and non-invasive imaging tool to detect signs of GCA. After more than 25 years since it was first described by Schmidt et al. [3], US has displaced TA biopsy being nowadays the preferred method to detect GCA. Moreover, the recently published European League Against Rheumatism (EULAR) guidelines recommends TA and axillary arteries (AA) US exam as the first-line imaging test in centers with expertise when patients are presenting with cranial symptoms of GCA [4]. The diagnosis of GCA can be confirmed with US positive finding not requiring additional complementary exams. The halo sign is the most important US finding in GCA and it is defined as a homogeneous, hypoechoic wall thickening, well delineated towards the luminal side, visible in two perpendicular planes, most commonly concentric in transverse scan [5]. A recent meta-analysis by EULAR showed a pooled sensitivity of 77% and specificity of 96% for the halo sign [6]. Furthermore, the compression sign, which is defined as a thickened arterial wall that remains visible upon compression, is also useful for GCA screening [5].

In recent years, very high-resolution transducers have been available for the US exam providing new tools to assess GCA patients as the measurement of the intima-media thickness (IMT) by B-Mode without Doppler. In this context, cut-off values for TA and AA have been published, with excellent sensitivities and specificities (> 97%) for the diagnosis of GCA in subjects with a high pre-test probability [7–9]. More recently, the Halo Score has been proposed as a quantitative measure to determine the extent of vascular inflammation by US, incorporating the IMT of each halo [10]. According to these findings, the Halo Score correlates with systemic markers of inflammation and risk for ocular ischemia, and a high Halo Score (≥ 10) may help to confirm GCA diagnosis with high specificity (> 95%). This novel scoring system has also been validated in routine care showing good diagnostic accuracy [11, 12].

However, IMTs above the cut-off values that mimic a positive halo and compression sign have been also described in patients not having GCA [13] leading to false positives in patients with atherosclerosis [14], intimal hyperplasia on biopsy [15], or in several disorders as amyloidosis [16], vasculitis mimics, or lymphoma [17]. Thus, the trend towards the utilization of IMT cut-off values to diagnose GCA needs to be evaluated in the clinical context of patients with elevated cardiovascular risk (CVR). It has been described how a positive compression sign or IMTs above the traditional cut-off values may be present in patients without GCA in the presence of atherosclerosis or elevated CVR [15]. Besides, quantitative US tools like the Halo Score, relying on the measurement of each artery IMT, may be influenced by the CVR, so an accurate interpretation of the score needs to be taken into account in this context.

The primary objective of this study was to evaluate the impact of CVR on the diagnostic accuracy of the US Halo Score in patients with suspected GCA.

## Methods

### Patients

This was a retrospective cross-sectional study including patients referred to a US fast track clinic (FTC) [2] at our Academic Center for screening of possible GCA over a 2-year period (from June 2019 to June 2021). Patients with suspected GCA are referred to this US clinic for examination within 24 h per protocol. For the purposes of this study, only consecutive patients with GCA suspicion were included. The study was performed in routine clinical practice conditions.

### Data collection

The following variables were collected from the electronic health records: demographics, presenting symptoms (headache, scalp tenderness, jaw claudication, visual symptoms and ocular ischemia diagnosis by an ophthalmologist, fever, polymyalgia rheumatica, and constitutional symptoms), previous use of glucocorticoids, and laboratory variables as C-reactive protein (CRP), erythrocyte sedimentation rate (ESR), hemoglobin, and platelets. TA biopsy data were also included if available. The gold standard for GCA diagnosis was the clinical confirmation by the treating clinician after a 6 months follow-up. The clinical GCA diagnosis could be dismissed according to the clinician criteria, even in patients fulfilling ACR 1990 criteria or with positive imaging tests, if another more reasonable diagnosis was established.

### Cardiovascular risk stratification

To assess the CVR, the following variables were collected: body weight, height and body mass index, history of acute myocardial infarction, acute coronary syndromes, transient ischemic attack, stroke, aortic aneurism, peripheral artery disease, diabetes mellitus with or without organ damage, estimated glomerular filtration rate (eGFR) using the Chronic Kidney Disease Epidemiology Collaboration (CKD-EPI) formula, cholesterol level (total cholesterol, high-density lipoprotein cholesterol and low-density lipoprotein cholesterol), systolic and diastolic blood pressure, and smoking habit (current or previous smoker). The European Society of Cardiology (ESC) Guidelines on CV Disease Prevention in clinical practice were applied to define four different categories of CVR [18]. We calculated the risk score of each patient using the available ESC CVD Risk Calculator app for mobile devices (https://www.escardio.org/Education/ESC-Prevention-of-CVD-Programme/Risk-assessment/esc-cvd-risk-calculation-app), and subjects were classified as very high, high, moderate, or low CVR. Patients with high or very high CVR were compared with patients with low or moderate CVR to determine differences in the diagnostic accuracy of the Halo Score.

### Ultrasound assessment

The three TA segments (common superficial TA, its parietal and frontal branches) and extracranial (carotid, subclavian and distal axillary) arteries were bilaterally evaluated by US in all patients within 24 h per protocol (excluding weekends with delays up to 48 h). The exam was performed in a supine position, by a single experienced ultrasonographer (JMC) using an EsaoteMyLab8 (Esaote, Genoa) with a hockey stick 12–18 MHz high frequency transducer for the temporal arteries and an 8–14 frequency transducer for extracranial arteries. The distal axillary arteries were scanned from the axillary fossa. The focus was positioned at 5 mm below the skin for the TA and 2–3 cm for the axillary arteries. The pulse repetition frequency was 2–3 kHz. The color box was set at an angle between sound waves and artery < 60°. The presence or absence of a halo, compression, stenosis, or occlusion for cranial arteries and the presence of a halo for extracranial arteries were evaluated. The IMT was measured in gray scale mode in all the arteries included in the protocol if technically possible due to the maximum frequency probe (18 MHz). Doppler color mode was only used to better delineate the lumen of the vessel to improve the visibility of the IMT in unclear cases. The extent of vascular inflammation was quantified according to the Halo Score, identifying the maximum IMT in each artery and calculating the composite score according to predefined cut-off values. The halo grade scores of the axillary arteries were multiplied by a factor of 3. The Halo score values could range from 0 to 48 [10]. The presence of a halo and/or compression sign in temporal arteries or the presence of a halo in extracranial arteries in the absence of atherosclerosis was considered sufficient for a positive US examination. The ultrasonographer was not blinded to the clinical information of the patient.

### Statistical analysis

Quantitative data were described as mean (standard deviation, SD) and qualitative variables as absolute frequency (percentages). Chi-square test or Fisher’s exact test were used to analyze differences between proportions; Student’s *t* test was used for comparison between means. Criterion validity was evaluated using receiver operating characteristic (ROC) curves with GCA clinical diagnosis as external criterion and construct validity was determined by Spearman’s rank correlation coefficient (rho). All tests were two-sided; *p* values < 0.05 were considered statistically significant. The SPSS software (version 23.0; IBM, USA) was used for statistical analysis.

### Ethical approval

This study was performed in accordance with the ethical standards of the responsible committee on human experimentation and the Helsinki Declaration of 1975, as revised in 1983. Research ethics committee approval for the protocol was obtained prior to commencing the study (RHEUM0322). The committee determined that written informed consent was not mandatory.

## Results

### Patient characteristics

**Fig. 1:**
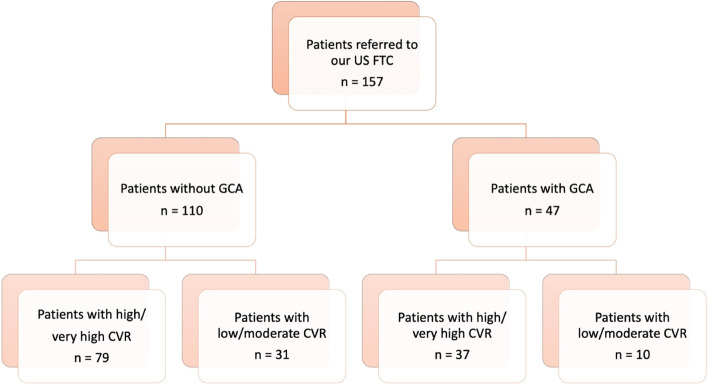
Flowchart of patients included in the FTC. FTC, fast track clinic; US, ultrasound; GCA, giant cell arteritis

A total of 157 patients referred to the FTC were included for analysis; 106 (67.5%) were female with a mean age of 73.7 years. Figure 1 shows the patient flow diagram. After 6 months of follow-up, 47 (29.9%) patients were clinically diagnosed of GCA by the treating clinician. Polymyalgia rheumatica diagnosis before US examination was present in 43 (27.4%) patients. Table 1 summarizes the baseline characteristics, clinical, laboratory and imaging findings of patients with and without GCA. Patients with GCA presented higher values of CRP, ESR, and platelets. TA biopsy was performed per clinician criteria in 31 patients with positive results in 10 (43.5%) GCA patients.

**Table 1:** Clinical, laboratory variables, and US findings in patients with or without GCA

	Total ***n*** = 157	Patients with GCA ***n*** = 47 (29.9)	Patients without GCA ***n*** = 110 (70.1)	***p***
**Demographics**				
**Age, mean (SD)**	73.7 (10.8)	75.3 (11.3)	73 (10.6)	0.245
**Female,** ***n*** **(%)**	106 (67.5)	31 (66)	75 (68.2)	0.785
**Clinical variables**				
**Baseline use of steroids,** ***n*** **(%)**	78 (50)	21 (45.7)	57 (51.8)	0.482
**PMR diagnosis before US examination,** ***n*** **(%)**	43 (27.4)	8 (17)	35 (31,8)	0.121
**Body mass index, mean (SD)**	27.2 (4.5)	26.8 (2.8)	27.4 (5.1)	0.415
**Systolic blood pressure, mean (SD)**	128.3 (16.8)	127.7 (19)	128.6 (15.9)	0.777
**Diastolic blood pressure, mean (SD)**	72.9 (10.6)	69.4 (8.6)	74.4 (11)	0.003
**Hypertension,** ***n*** **(%)**	96 (61.5)	30 (65.2)	66 (60)	0.541
**Acute myocardial infarction,** ***n*** **(%)**	6 (3.8)	1 (2.2)	5 (4.5)	0.482
**Arterial revascularization,** ***n*** **(%)**	10 (6.4)	4 (8.7)	6 (5.5)	0.451
**Transient ischemic attack,** ***n*** **(%)**	4 (2.6)	0 (0)	4 (3.7)	0.188
**Acute ischemic stroke,** ***n*** **(%)**	12 (7.7)	3 (6.5)	9 (8.2)	0.723
**Peripheral artery disease,** ***n*** **(%)**	2 (1.3)	1 (2.2)	1 (0.9)	0.522
**Diabetes mellitus type 2,** ***n*** **(%)**	37 (23.7)	13 (28.3)	24 (21.8)	0.388
**Diabetes mellitus with target organ damage,** ***n*** **(%)**	4 (2.6)	1 (2.2)	3 (2.7)	0.842
**Chronic kidney disease,** ***n*** **(%)**	28 (17.9)	6 (13)	22 (20)	0.302
**Glomerular filtration rate (MDRD), mean (SD)**	84.4 (23.4)	84.1 (22.5)	84.5 (23.8)	0.931
**Current or previous smokers,** ***n*** **(%)**	41 (26.3)	16 (34.8)	25 (22.7)	0.119
**Very high CVR**	70 (44.6)	23 (48.9)	47 (42.7)	0.473
**High CVR**	46 (29.3)	14 (29.8)	32 (29.1)	0.93
**Moderate CVR**	21 (13.4)	6 (12.8)	15 (13.6)	0.883
**Low CVR**	20 (12.7)	4 (8.5)	16 (14.5)	0.299
**SCORE Score, mean (SD)**	19.2 (21.2)	20.6 (21.6)	18.7 (21)	0.601
**Laboratory findings**				
**CRP (mg/dL), mean (SD)**	5.9 (11.3)	10.7 (18.2)	3.8 (5)	0.001
**ESR (mm/h), mean (SD)**	52.8 (34.2)	68.2 (34)	45 (31.8)	0.001
**Hemoglobin (g/dL), mean (SD)**	13.7 (17.6)	11.7 (1.6)	14.5 (21)	0.185
**Platelets 10**^**9**^**/L, mean (SD)**	293 (124.7)	335.4 (143.3)	274.7 (111.6)	0.014
**Total cholesterol mg/dL, mean (SD)**	181.9 (153.3)	161.3 (42.6)	190.6 (180.1)	0.111
**HDL cholesterol mg/dL, mean (SD)**	54 (18.7)	53.1 (20.7)	54.3 (17.8)	0.723
**LDL cholesterol mg/dL, mean (SD)**	97.4 (35.9)	90.8 (30.9)	100.1 (37.6)	0.142
**Histology**				
**Positive temporal artery biopsy (*n*** **= 31),** ***n*** **(%)**	10 (32.3)	10 (43.5)	0 (0%)	0.023
**US variables**				
**Positive US findings,** ***n*** **(%)**	46 (29.3%)	41 (87.2%)	5 (4.5%)	< 0.001
**Temporal artery positive US findings,** ***n*** **(%)**	32 (20.4%)	29 (61.7%)	3 (2.7%)	< 0.001
**Extracranial arteries positive US findings,** ***n*** **(%)**	23 (14.6%)	21 (44.7%)	2 (1.8%)	< 0.001
**Temporal + extracranial arteries positive US findings,** ***n*** **(%)**	9 (5.7%)	9 (19.1%)	0 (0%)	< 0.001
**Halo Score, mean (SD)**	11.4 (8.3)	18.2 (9.2)	8.5 (5.9)	< 0.001

*Abbreviations:* *GCA*, giant cell arteritis; *PMR*, polymyalgia rheumatica; *CRP*, C-reactive protein; *ESR*, erythrocyte sedimentation rate; *US*, ultrasound; *HDL*, high-density lipoprotein; *LDL*, low-density lipoprotein; *SD*, standard deviation

### Cardiovascular risk assessment

According to the ESC Guidelines on CV Disease Prevention, 70 (44.6%) patients were classified as very high CVR, 46 (29.3%) as high CVR, 21 (13.4%) as moderate CVR, and 20 (12.7%) as low CVR. Overall, mean score SCORE was 19.2 (21.2). There were no differences in CVR between patients with and without GCA (mean SCORE 20.6 [21.6] vs 18.7 [21]; *p* = 0.601) (Table 1).

### Ultrasound findings

Patients with GCA presented positive US findings more frequently than subjects without GCA (41 [87.2%] vs 5 [4.5%]; *p* < 0.001). Sensitivity and specificity of US vs GCA clinical confirmation was 87.2% and 95.5%, respectively, and positive and negative likelihood ratio was 19.38 and 0.13, respectively. Among patients with GCA, 29 (61.7%) had TA involvement and 21 (44.7%) had extra-cranial arteries inflammation according to US examination. A mixed pattern with involvement of both TA and extracranial arteries was found in 9 (19.1%) patients. Twenty (42.6%) patients had exclusive cranial involvement, while 12 (25.5%) had exclusive extracranial involvement. Mean Halo Score was significantly higher in patients with GCA (18.2 (9.2) vs 8.5 (5.9); *p* < 0.001) (Table 1).

### Impact of cardiovascular risk on the intima-media thickness of cranial and extracranial arteries

Among patients without GCA, extra-cranial artery IMT was significantly higher in patients with high/very high CVR than in those with low/moderate CVR (carotid IMT 0.83 (0.16) vs 0.74 (0.13); *p* < 0.001, subclavian IMT 0.74 (0.18) vs 0.6 (0.13); *p* < 0.001 and axillary IMT 0.71 (0.16) vs 0.57 (0.15); *p* < 0.001) (Table 2). Similarly, numerically higher IMT values were found in TA in patients with high/very high CVR vs low/moderate CVR in patients without GCA but without statistical significance.

**Table 2:** Measurements of IMT in cranial and extracranial arteries and Halo Score values according to CVR

	Total ***n*** = 157	Patients with GCA ***n*** = 47			Patients without GCA ***n*** = 110		
		Patients with high/very high CVR ***n*** = 37 (78.7%)	Patients with low/moderate CVR ***n*** = 10 (21.3%)	***p***	Patients with high/very high CVR ***n*** = 79 (71.8%)	Patients with low/moderate CVR ***n*** = 31 (28.2%)	***p***
**Superficial temporal artery (right) mm, mean (SD)**	0.43 (0.21)	0.74 (0.26)	0.39 (0.02)	0.094	0.34 (0.06)	0.31 (0.07)	0.320
**Superficial temporal artery (left) mm, mean (SD)**	0.44 (0.19)	0.58 (0.23)	0.52 (0.15)	0.695	0.35 (0.12)	0.35 (0.07)	0.994
**Superficial temporal artery (both) mm, mean (SD)**	0.43 (0.2)	0.66 (0.25)	0.45 (0.11)	0.025	0.35 (0.09)	0.32 (0.07)	0.354
**Frontal branch (right) mm, mean (SD)**	0.3 (0.12)	0.41 (0.18)	0.34 (0.17)	0.37	0.25 (0.04)	0.26 (0.07)	0.401
**Frontal branch (left) mm, mean (SD)**	0.3 (0.12)	0.42 (0.18)	0.28 (0.14)	0.096	0.27 (0.05)	0.26 (0.04)	0.832
**Frontal branch (both) mm, mean (SD**	0.3 (0.12)	0.42 (0.18)	0.31 (0.15)	0.056	0.26 (0.05)	0.26 (0.06)	0.577
**Parietal branch (right) mm, mean (SD)**	0.32 (0.13)	0.45 (0.19)	0.33 (0.1)	0.125	0.27 (0.04)	0.28 (0.07)	0.504
**Parietal branch (left) mm, mean (SD)**	0.31 (0.12)	0.41 (0.16)	0.39 (0.16)	0.006	0.27 (0.06)	0.28 (0.09)	0.288
**Parietal branch (both) mm, mean (SD)**	0.32 (0.12)	0.43 (0.17)	0.35 (0.12)	0.102	0.27 (0.04)	0.28 (0.08)	0.173
**Carotid artery (right) mm, mean (SD)**	0.82 (0.21)	0.85 (0.17)	1 (0.54)	0.112	0.83 (0.17)	0.72 (0.14)	0.002
**Carotid artery (left) mm, mean (SD)**	0.88 (0.28)	0.92 (0.25)	1.2 (0.6)	0.310	0.84 (0.15)	0.76 (0.12)	0.006
**Carotid artery (both) mm, mean (SD)**	0.85 (0.25)	0.88 (0.21)	1.2 (0.6)	< 0.001	0.83 (0.16)	0.74 (0.13)	< 0.001
**Subclavian artery (right) mm, mean (SD)**	0.81 (0.3)	0.9 (0.34)	1.2 (0.6)	0.845	0.78 (0.17)	0.62 (0.14)	< 0.001
**Subclavian artery (left) mm, mean (SD)**	0.73 (0.26)	0.81 (0.26)	1.1 (0.45)	0.901	0.71 (0.18)	0.58 (0.13)	< 0.001
**Subclavian artery (both) mm, mean (SD)**	0.77 (0.28)	0.86 (0.31)	1.2 (0.5)	0.001	0.74 (0.18)	0.6 (0.13)	< 0.001
**Axillary artery (right) mm, mean (SD)**	0.78 (0.34)	0.93 (0.41)	1.2 (0.72)	0.268	0.72 (0.16)	0.6 (0.15)	0.001
**Axillary artery (left) mm, mean (SD)**	0.77 (0.34)	0.92 (0.36)	1.23 (0.78)	0.0.92	0.71 (0.16)	0.57 (0.15)	< 0.001
**Axillary artery (both) mm, mean (SD)**	0.77 (0.34)	0.92 (0.38)	1.22 (0.73)	0.021	0.72 (0.16)	0.59 (0.15)	< 0.001
**Halo Score, mean (SD)**	11.39 (8.3)	18.5 (8.8)	17.2 (10.6)	0.69	9.38 (5.93)	6.16 (5.22)	0.007

*SD*, standard deviation

Regarding the GCA group, patients with high/very high CVR had significantly higher IMT measurements in superficial TA (0.66 [0.25] vs 0.45 [0.11]; *p* = 0.025) and numerically higher IMT values in frontal and parietal branches (0.42 (0.18) vs 0.31 (0.15); *p* = 0.056 and 0.43 (0.17) vs 0.35 (0.12); *p* = 0.102, respectively). GCA patients showed significantly higher IMT values in patients with low/moderate CVR than in those with high/very high CVR (carotid IMT 0.88 (0.21) vs 1.2 (0.6); *p* < 0.001, subclavian IMT 0.86 (0.31) vs 1.2 (0.5); *p* = 0.001 and axillary IMT 0.92 (0.38) vs 1.22 (0.73); *p* = 0.021). However, patients with GCA and low/moderate CVR were significantly younger (58.4 vs 79.9; *p* < 0.001) and had extracranial artery involvement more frequently (70% vs 37.8%; *p* = 0.07) compared with those with GCA and high/very high CVR.

### Impact of cardiovascular risk on the diagnostic accuracy of the Halo Score

**Fig. 2:**
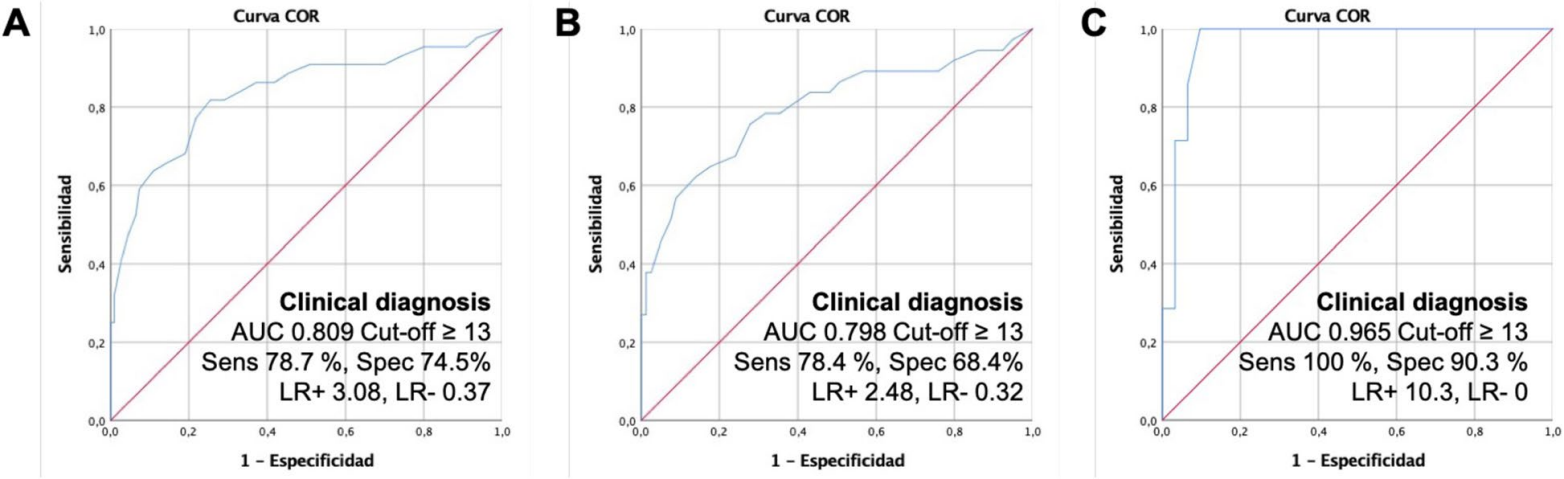
Diagnostic accuracy of the Halo Score for a clinical diagnosis of GCA after 6 months follow-up in **A** all LVV suspected patients, **B** patients with high to very high CVR, and **C** patients with low to moderate CVR. Youden index was used to determine the optimal cut-off points. AUC, area under the curve, LR+, positive likelihood ratio; LR−, negative likelihood ratio; ROC, receiver operating characteristic; Sens, sensitivity; Spec, specificity

**Fig. 3:**
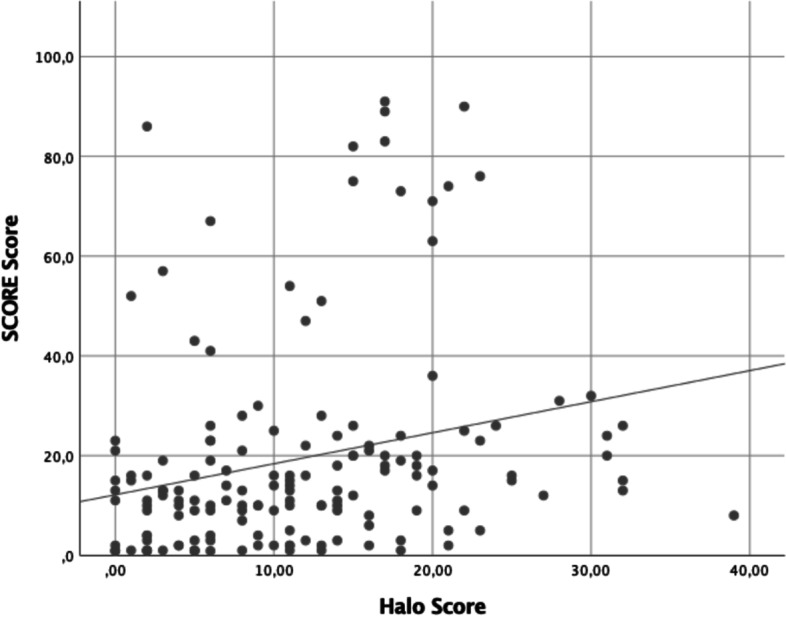
Correlation between the Halo Score and the SCORE score (*r* 0.245; *p* = 0.002)

The Halo Score values were significantly higher in non-GCA patients with high/very high CVR vs low/moderate CVR (9.38 (5.93) vs 6.16 (5.22); *p* = 0.007) (Table 2). The area under the ROC curve of the Halo Score to identify GCA was 0.835 (95% CI 0.756–0.914), slightly greater in patients with low/moderate CVR (0.965 [95% CI 0.911–1]) versus patients with high/very high CVR (0.798 [95% CI 0.702–0.895]) (Fig. 2). A statistically weak positive correlation was found between the Halo Score and the SCORE (*r* 0.245; *p* = 0.002) (Fig. 3).

## Discussion

In this observational cross-sectional study, we evaluated for the first time the impact of the CVR evaluation on the diagnostic accuracy of the most used US quantitative index, the Halo Score. Our main finding is that patients with high and very high CVR show higher US Halo Score affecting the diagnostic accuracy of this US index.

IMT cut-off values for GCA diagnosis have been previously described [7–9, 19], but the impact of the individually CVR on the diagnostic accuracy of US has not been well determined. Similar to our results, De Miguel et al evaluated the IMT of carotid and TA in 40 patients with high CVR [14]. They found that atherosclerotic disease with a carotid IMT > 0.9 mm increases the IMT of TA and might mimic the halo sign. They proposed a cut-off of TA IMT > 0.34 mm in at least two branches to minimize false positives in a GCA diagnosis, achieving high specificity (> 95%). In this study, the authors did not evaluate the impact of atherosclerosis or CVR in the extracranial (p.e axillary) artery IMT. More recently, Martire et al. [20] investigated the performance of previously proposed IMT cut-off values [7], scanning the temporal and axillary arteries in a cohort of non-GCA. They found that CVR was associated with higher IMT values, especially at superficial temporal artery level, showing IMT values higher than the proposed cut-off values in 21% of healthy subjects with high/very high CVR, and in only 3.2% of subjects with low/moderate CVR.

To our knowledge, this is the first study specifically design to evaluate the diagnostic performance of the US Halo Score for GCA in different CVR subgroups of patients. According to our findings, the diagnostic accuracy of the US Halo Score is reduced in patients with high/very high CVR in comparison with patients with low/moderate CVR. A possible explanation is that the Halo Score is based on the measurement of the halo (or increased IMT), which would be influenced by the CVR of each individual patient. Our results are in line with other previously published by Martire et al. [20], although they studied a slightly different population (healthy subject without GCA or PMR), in contrast with a suspected GCA population in our cohort. In agreement with de Miguel et al. results [14], a numerically higher IMT of TA were found in patients with high/very high CVR, although we did not specifically include atherosclerosis in our analysis. Subjects without GCA with high/very high CVR had numerically higher IMT of TA and significantly higher IMT of extracranial arteries than those with low/moderate CVR (Table 2). Similarly, GCA patients with high/very high CVR had higher IMT of TA. Surprisingly, among patients with GCA, IMT of extracranial arteries was higher in those with low/moderate CVR. However, these results are explained by the fact that patients with GCA and extracranial involvement are significantly younger than those with TA involvement and, therefore, they had lower CVR. Our results highlight the importance of appropriate interpretation of IMT values when assessing patients with suspected GCA. The ultrasonographer should be cautious when assessing patients for GCA screening and be able to discard the disease even with IMT values above the diagnostic cut-offs if patients have a low pre-test probability and elevated CVR.

The main strength of the present study is that it is the first to evaluate the impact of CVR on the diagnostic accuracy of the Halo Score for GCA diagnosis, in a relatively large cohort of patients with suspected GCA evaluated in a well stablished FTC. This study has several limitations including the retrospective design and the limited data for some ancillary studies as TAB which was only performed per clinician criteria, consistent with real-world clinical practice. Second, the ultrasonographer was not blinded to clinical data. Third, intra and inter-observer reliability was not investigated.

In summary, high and very high CVR may influence the diagnostic accuracy of the US Halo Score leading to false-positive findings in these patients. Higher IMT values may be found in cranial and extracranial arteries in subjects with high/very high CVR without GCA. Thus, CVR should be taken into consideration in the US vascular assessment of patients with suspected GCA. These results need to be confirmed in additional cohorts to determine the need of a modified US Halo Score in patients with high and very high CVR.

## Data Availability

The data underlying this article are available in the article and in its online supplementary material.

## References

[1] Jennette JC, Falk RJ, Bacon PA, Basu N, Cid MC, Ferrario F (2013). 2012 revised International Chapel Hill Consensus Conference Nomenclature of Vasculitides. Arthritis Rheum.

[2] Diamantopoulos AP, Haugeberg G, Lindland A, Myklebust G (2016). The fast-track ultrasound clinic for early diagnosis of giant cell arteritis significantly reduces permanent visual impairment: towards a more effective strategy to improve clinical outcome in giant cell arteritis?. Rheumatology (Oxford).

[3] Schmidt WA, Kraft HE, Vorpahl K, Völker L, Gromnica-Ihle EJ (1997). Color duplex ultrasonography in the diagnosis of temporal arteritis. N Engl J Med.

[4] Dejaco C, Ramiro S, Duftner C, Besson FL, Bley TA, Blockmans D (2018). EULAR recommendations for the use of imaging in large vessel vasculitis in clinical practice. Ann Rheum Dis.

[5] Chrysidis S, Duftner C, Dejaco C, Schäfer VS, Ramiro S, Carrara G (2018). Definitions and reliability assessment of elementary ultrasound lesions in giant cell arteritis: a study from the OMERACT Large Vessel Vasculitis Ultrasound Working Group. RMD Open.

[6] Duftner C, Dejaco C, Sepriano A, Falzon L, Schmidt WA, Ramiro S (2018). Imaging in diagnosis, outcome prediction and monitoring of large vessel vasculitis: a systematic literature review and meta-analysis informing the EULAR recommendations. RMD Open.

[7] Schäfer VS, Juche A, Ramiro S, Krause A, Schmidt WA (2017). Ultrasound cut-off values for intima-media thickness of temporal, facial and axillary arteries in giant cell arteritis. Rheumatology (Oxford).

[8] Ješe R, Rotar Ž, Tomšič M, Hočevar A (2021). The cut-off values for the intima-media complex thickness assessed by colour Doppler sonography in seven cranial and aortic arch arteries. Rheumatology (Oxford).

[9] Czihal M, Schröttle A, Baustel K, Lottspeich C, Dechant C, Treitl KM (2017). B-mode sonography wall thickness assessment of the temporal and axillary arteries for the diagnosis of giant cell arteritis: a cohort study. Clin Exp Rheumatol.

[10] van der Geest KSM, Borg F, Kayani A, Paap D, Gondo P, Schmidt W (2020). Novel ultrasonographic Halo Score for giant cell arteritis: assessment of diagnostic accuracy and association with ocular ischaemia. Ann Rheum Dis.

[11] Molina Collada J, Martínez-Barrio J, Serrano-Benavente B, et al. Diagnostic value of ultrasound halo count and Halo Score in giant cell arteritis: a retrospective study from routine care. Ann Rheum Dis. 2022;81:e175. 10.1136/annrheumdis-2020-218631 32759266

[12] van der Geest KS, Dasgupta B. Response to: ‘Diagnostic value of ultrasound halo count and Halo Score in giant cell arteritis: a retrospective study from routine care’ by Molina Collada et al. Ann Rheum Dis. 2022;81:e176. 10.1136/annrheumdis-2020-218654 32759255

[13] Schmidt WA (2019). The ultrasound halo sign of temporal arteries: is it always giant cell arteritis?. Rheumatology (Oxford).

[14] De Miguel E, Beltran LM, Monjo I, Deodati F, Schmidt WA, Garcia-Puig J (2018). Atherosclerosis as a potential pitfall in the diagnosis of giant cell arteritis. Rheumatology (Oxford).

[15] Seitz L, Lötscher F (2021). The intima-media thickness in suspected giant cell arteritis-sometimes it is worth taking a closer look. Rheumatology (Oxford).

[16] Molina Collada J, Ruíz Bravo-Burguillos E, Monjo I, Bonilla G, Fernández E, Balsa A (2019). Positive ultrasound halo sign of temporal arteries due to amyloidosis. Rheumatology (Oxford).

[17] Fernández-Fernández E, Monjo-Henry I, Bonilla G, Plasencia C, Miranda-Carús ME, Balsa A (2020). False positives in the ultrasound diagnosis of giant cell arteritis: some diseases can also show the halo sign. Rheumatology (Oxford).

[18] Visseren FLJ, Mach F, Smulders YM, Carballo D, Koskinas KC, Bäck M (2021). ESC Guidelines on cardiovascular disease prevention in clinical practice. Eur J Prev Cardiol.

[19] López-Gloria K, Castrejón I, Nieto-González JC, Rodríguez-Merlos P, Serrano-Benavente B, González CM (2022). Ultrasound intima media thickness cut-off values for cranial and extracranial arteries in patients with suspected giant cell arteritis. Front Med (Lausanne).

[20] Martire MV, Cipolletta E, Di Matteo A, Di Carlo M, Jesus D, Grassi W (2021). Is the intima-media thickness of temporal and axillary arteries influenced by cardiovascular risk?. Rheumatology (Oxford).

